# Health-related quality of life in patients treated with en bloc resection for primary tumors of the spine

**DOI:** 10.3389/fonc.2024.1485226

**Published:** 2024-11-20

**Authors:** Luigi Emanuele Noli, Chiara Alcherigi, Cristiana Griffoni, Eleonora Pesce, Simona Rosa, Gisberto Evangelisti, Valerio Pipola, Paolo Francesco Davassi, Annalisa Monetta, Giovanni Barbanti Brodano, Silvia Terzi, Riccardo Ghermandi, Giuseppe Tedesco, Marco Girolami, Stefano Bandiera, Alessandro Gasbarrini

**Affiliations:** ^1^ ISNB Istituto delle Scienze Neurologiche di Bologna, Bologna, Italy; ^2^ Department of Spine Surgery, IRCCS Istituto Ortopedico Rizzoli, Bologna, Italy; ^3^ Department of Biomedical and Neuromotor Sciences, Alma Mater Studiorum University of Bologna, Bologna, Italy

**Keywords:** primary spine tumors, en bloc resection, health-related quality of life, adverse events, mental component, physical component

## Abstract

**Study design:**

Retrospective analysis

**Objective:**

The aim of this study is to evaluate the functional outcomes and the health-related quality of life (HRQOL) in patients undergoing en bloc resection of spinal tumor.

**Summary of background data:**

En bloc resection in the spine is a surgical procedure designed to completely remove a tumor in one piece, with wide margins preserved, in order to reduce the risk of local recurrences. This demanding procedure has been shown to improve local control and survival rate, besides a relatively high morbidity.

**Methods:**

Between 2016 and 2021, 70 patients underwent en bloc resection surgery for a primary spine tumor and 38 came out to be eligible for this analysis. Eligibility criteria include at least one follow-up visit within a two-year period from surgery and Patient Reported Outcomes evaluation collected prospectively at baseline and at least one follow- up in the range 4-24 months. The outcome variables (EQ5D Numeric Scale and Index, SF36 scores and NRS score) were analyzed with multilevel linear mixed-effects regression. Baseline- (age, gender, localization, histotype, number of levels of resection, previous surgery) and time-dependent covariates (adverse events, spinal cord damage) were included.

**Results:**

Beside a slight improvement of all the scores, no significant differences were found between baseline and follow up times for EQ-5D-3L Numeric Scale and Index and for SF-36 Standardized Physical component. SF-36 Standardized Mental component appeared to be significantly better at 12-month FU compared to baseline. Ultimately, age over 50 years old and the occurrence of adverse events emerged to be as the two main factor determining worsening in several HRQOL scores. Pain came out to be significantly reduce at 24-month compared to baseline.

**Conclusions:**

The aim of en bloc resection in the treatment of primary spinal tumors is to improve survival rates and reduce local recurrences. Despite its radicality, our preliminary results suggest that patients experience a slight to moderate improvement postoperatively compared to their preoperative perceived health status.

## Introduction

Primary bone tumors of the spine are extremely rare, accounting for approximately 5% of all primary bone tumors (1). En bloc resection is a surgical technique designed to completely remove a tumor in one piece along with a surrounding layer of healthy tissue, preventing tumor injury and minimizing the risk of local recurrence (2–6). This surgical approach applied to spinal tumors was first proposed by Enneking et al (7), then pioneered by Stener (8), followed by advances by Roy-Camille et al (9)l, and subsequently by Tomita et al (3).

En bloc resection in the spine is typically recommended for aggressive benign tumors (stage 3), malignant primary tumors, and selected isolated metastases (10–12). Adjuvant therapies should be considered, especially for high-grade malignancies, to improve oncologic outcomes, but it should be mentioned that perioperative treatments may also contribute to local complications, including wound dehiscence and infection (13).

En bloc surgery for primary spinal tumors has been shown to guarantee better local control and survival rates compared to less aggressive surgery (4, 10, 14). In particular, previous literature for chordoma has demonstrated that en bloc resection with adequate margins, respecting Enneking Appropriateness (EA) criteria when clinically and anatomically possible, is able to improve patients’ survival and decrease local recurrence, with respect to patients treated with Enneking Inappropriate margins (15, 16), even if growing evidence has been collected about the important role that charged-particle therapy has in the treatment of spinal tumors in different settings, from the primary treatment of unresectable lesions to the neo-/adjuvant role (17–20).

En bloc resection is a demanding procedure associated with relatively high morbidity, ranging from 79% (21) to 48% (4) of patients experiencing at least one adverse event. In fact, en bloc resection requires extensive dissection of the spine and adjacent relevant structures, often adjacent to or invaded by the tumor (nerve roots, spinal cord, blood vessels) (3, 14), which may be damaged to achieve better surgical margins. Risks and adverse events are well known and range from bleeding, vascular injury, dural tears, neurological damage and other intraoperative events to postoperative complications such as mechanical instability, infection and wound related issues (4, 14, 22, 23).

Regarding primary bone tumors, advancements in radiation therapy and targeted drugs have significantly changed disease management, but surgical treatment remains crucial in determining the outcomes for many of these tumors.

In our previous study (4), based on the analysis of 298 patients who underwent en bloc resection in our Institution from 1980 to 2021, we found that the improved disease control achieved through out this surgery is associated with enhanced survival rates (75% at 5 years and 67% at 10 years), and that the high incidence of complications (reported in 48% of patients) does not adversely affect survival rates. However, there is a lack of evidence regarding the functional outcomes and health-related quality of life (HRQOL) in patients undergoing this radical resection.

The aim of this study is to provide new evidence on HRQOL, even if in a smaller cohort of patients undergoing en bloc resection for primary tumors of the spine, and identify risk factors that may contribute to functional impairment in these patients.

## Methods

This is a retrospective analysis of data prospectively collected as part of a registry approved by the Institutional Ethics Committee of Istituto Ortopedico Rizzoli on 14.12.2016, concerning the retrospective and prospective collection of clinical and radiographic data related to spinal diseases (of degenerative, oncological, traumatic and infectious origin) (protocol number 0022814).

The research was performed according to the Declaration of Helsinki. The signature of a study-specific informed consent was obtained from patients prospectively enrolled for the study, but it was not required for retrospective data due to the regulations taking place in health institutions dedicated to scientific research.

Potentially eligible patients were retrospectively identified from medical records at the Rizzoli Institute, and their demographic and clinical data were extracted.

The eligibility criteria for this study included: en bloc surgery resection of a primary (benign or malignant) spine tumor; at least one follow-up visit within a two-year period from surgery; Patient Reported Outcomes (PROs) evaluation at baseline and at least one follow up in the range 4-24 months.

Between 2016 and 2021, 70 patients underwent en bloc resection surgery for a primary spine tumor. The surgical indication for en bloc resection was based on histology, staging (Enneking and WBB classifications), and patient’s clinical condition and prognosis. All patients were treated by the same team of surgeons.

**Figure 1:**
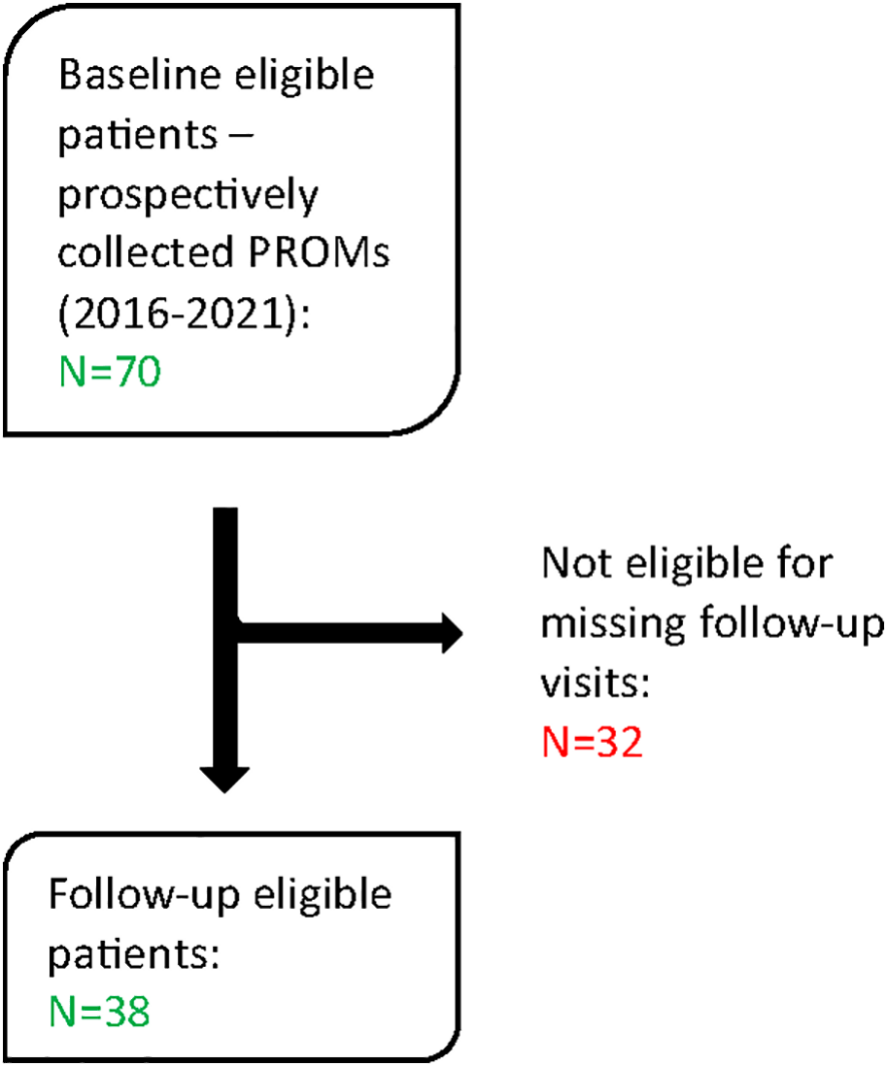
Patients’ flow-chart.

Of the initial cohort 32 patients were lost to follow-up and no PROMs were available to evaluate HRQOL. Ultimately, 38 patients met the eligibility criteria and were included in the present analysis (**Figure 1**).

To collect the PROs, three outcome measurements available in Italian language were used: EQ-5D-3L, Short-Form 36 Health Survey (SF-36) and the Numeric Rating Scale (NRS). EQ-5D-3L by EuroQol Group is a self-administered questionnaire that comprises the quality-of-life related dimensions of mobility, self-care, usual activities, pain/discomfort and anxiety/depression. Each dimension has 3 levels: no problems, some problems, and extreme problems and the patient is asked to indicate his/her health state. In addition to the 5 domains, a general Numeric Scale from 0 to 100 must be filled by patients, with 100 meaning the personal and perceived maximum health status). To have a comprehensive representation of EQ-5D-3L based on patients’ answers, an Index from -0.109 to 1.00 has been developed, with 1.00 representing the best possible outcome (24, 25).

SF-36 is a self-administered questionnaire consisting of 36 questions assessing various aspects of HRQOL, including vitality, physical functioning, bodily pain, general health perceptions, physical role functioning, emotional role functioning, social role functioning, and mental health or emotional well-being. Each closed-ended question yields a variable score contributing to the creation of two macro-domains: a Standardize Physical Component (SFC) and a Standardize Mental Component (SMC) scaled from 0 to 100. A score of 100 indicates the best possible health status (26, 27).

NRS is commonly used for measuring pain intensity in the neck, back, legs and arms and is well validated. It is scored from 0 to 10 (with 0 meaning no pain and 10 meaning the worst imaginable pain) (28).

Adverse events were prospectively collected and classified according to the Spinal Adverse Events Severity System (SAVES-V2) (29). Spinal cord injuries were classified according to the American Spinal Injury Association Impairment Scale (ASIA Score) (30).

### Statistical analysis

Quantitative variables were summarized as mean ± standard deviation; categorical variables were summarized as frequencies and percentages.

The outcome variables with 4 follow-up evaluations (EQ5D Numeric Scale and Index, SF36 scores and NRS score) were analyzed with multilevel linear mixed-effects regression.

*Post hoc* tests were performed to compare the outcomes scales respect different patient groups. The estimated mean scores are reported with 95% confidence interval.

The variable NRS pain scale was calculated for each follow-up as the maximum value among the patient’s imputed pain ratings of back, legs, arms, and neck.

Because there were multiple follow-up evaluations per patient, we fit a two-level model for each outcome with random intercepts at the patient level. Time was treated as a categorical covariate to examine possible nonlinear trends, which resulted in the inclusion of dummy variables for time in the model. The fixed portion of the model was then augmented by including, in addition to time dummies, age of patients (<50 or ≥50 years, mean age at surgery 45,9 years old), gender, Histotype (malignant *vs* benign), Vertebral levels removed (>1 *vs* 1), Previous surgery (yes/no), Complications (AEs yes/no) and ASIA Score (C-D *vs* E). To avoid sparse data, tumor localization was classified into two groups (lumbar/sacral *vs*. cervical/thoracic).

In addition, the study outcomes were modeled as a function of time-by-covariate interactions, which means that multiplication terms involving time dummies and covariates were included as further independent variables in the model. To avoid overfitting and ensure consistency, we constructed one multiplicative interaction term at a time and retained only age in the model (continuous variable), assessing the statistical significance of the interaction by means of the likelihood-ratio test. This approach was taken with the intention of investigating the presence of divergent posttreatment increments or decrements according to relevant baseline characteristics.

Because regression coefficient estimates are difficult to interpret when interactions are present, we opted for an indirect interpretation of the model via its predictions. More specifically, predicted means resulting from multilevel models were displayed using connected line charts, and differences (i.e., contrasts) across estimates at each follow-up evaluation were tested with the delta method.

The occurrence of surgical complications and the presence of neurological damages were incorporated in the mixed model as time-varying covariates to elucidate their impact on the HRQOL and capture their dynamic changes. The mixed model accounted for adverse events as they occurred, without categorizing them into intraoperative and postoperative.

Given the potential impact of neurological damages on quality of life and their time-varying nature, we included the ASIA scale as a time-dependent variable in our analysis. Patients were categorized into two groups: those with some degree of spinal cord injury (ASIA A, B, C, D) and those without damage (ASIA E).

Missing data followed an intermittent (monotonic) pattern rather than a dropout (attrition) pattern. By examining whether the outcomes at a specific time point (with complete data) were correlated with missing for that variable at the following time point (Little, 1995), no evidence against the missing-at-random (MAR) assumption was found. Therefore, no imputation was performed, and patients contributed to the model for the number of evaluations available during follow-up. Moreover, the likelihood-ratio test and normal Q–Q plot confirmed that level-one residual errors were homoscedastic and normally distributed.

All analyses were performed with Stata 17 (StataCorp. 2021. *Stata 17 Base Reference Manual.* College Station, TX: Stata Press). The significance level was set at 5%.

## Results

Based on eligibility criteria, 38 patients were included in this analysis. Most of patients had malignant primary tumors: 12 chordomas, 9 chondrosarcomas, 5 Ewing sarcomas, 3 hemangioendotheliomas, 1 hemangioma, 1 fibrosarcoma, and 1 osteosarcoma. Additionally, 6 patients had aggressive benign primary tumors, including 4 giant cell tumors and 2 osteoblastomas.

All surgical treatments in this cohort were Enneking Appropriate. Among all 38 patients, none died from the disease within the 24-month follow-up period. Four patients experienced disease progression, three of them showing metastatic progression and one suffering from recurrence.

12 patients completed PROMs questionnaires at 4 months follow up, 20 patients at 1 year, and 17 patients at 2 years.

Demographics and clinical data at baseline are reported in **Table 1**.

**Table 1:** Demographics and clinical data.

Age at surgery (mean, SD):		49,5 ± 17,6
**Male (N, %):**		22 (57,9)
**Tumor localization (N, %)**	Cervical, Thoracic	21 (55,3)
	Lumbar, Sacral	17 (44,7)
**Histotype (N, %)**	benign	9 (23,7)
	malignant	29 (76,3)
**Previous surgery (N, %)**	Yes	7 (18,4)
	No	31 (81,6)
**Vertebral levels removed (N, %)**	1	30 (78,9)
	>1	8 (21,1)
**ASIA Scale (N,%)**	ASIA E	20 (70,6)
	ASIA C-D	10 (29,4)
	N/A	4 (10,5)

As shown in **Table 2**, 9 patients (23.7%) experienced 1 intraoperative adverse event (8 out of 9 were classified as <3 severity grade, requiring minor invasive or no treatment at all). The most represented intraoperative AEs were dural tears (3/9), hardware malpositioning requiring revision (2/9) and visceral injury (2/9). 13 patients suffered at least 1 postoperative adverse event prior to the 4-month visit; 10 patients between 4- and 12-month visit; 5 patients between 12- and 24-month visit. A total of 5 postoperative AEs required invasive treatments (≥4 severity grade). Most of these postoperative AEs were categorized as “other”; 9 were construct failure without loss of correction; 4 were construct failure requiring a revision surgery.

**Table 2:** Adverse events.

		Patient with Intraop AE (N=9, 23.7%)		Patient with Postop AE (0-4 months) (N=13, 34.2%)		Patient with Postop AE (4-12 months) (N=10, 26,3%)		Patient with Postop AE (12-24 months) (N=5, 13.2%)	
		N	%	N	%	N	%	N	%
Number of adverse events	1	9	100,0%	9	69,2%	8	80,0%	4	80,0%
	2	0	0,0%	4	30,8%	1	10,0%	1	20,0%
	3	0	0,0%	0	0,0%	1	10,0%	0	0,0%
Grade of adverse events	1	0	0,0%	0	0,0%	2	20,0%	1	20,0%
	2	3	33,3%	4	30,8%	2	20,0%	0	0,0%
	3	5	55,6%	6	46,2%	4	40,0%	3	60,0%
	4	1	11,1%	2	15,4%	1	10,0%	1	20,0%
	5	0	0,0%	1	7,7%	0	0,0%	0	0,0%

### HRQOL trend in the population

**Figure 2:**
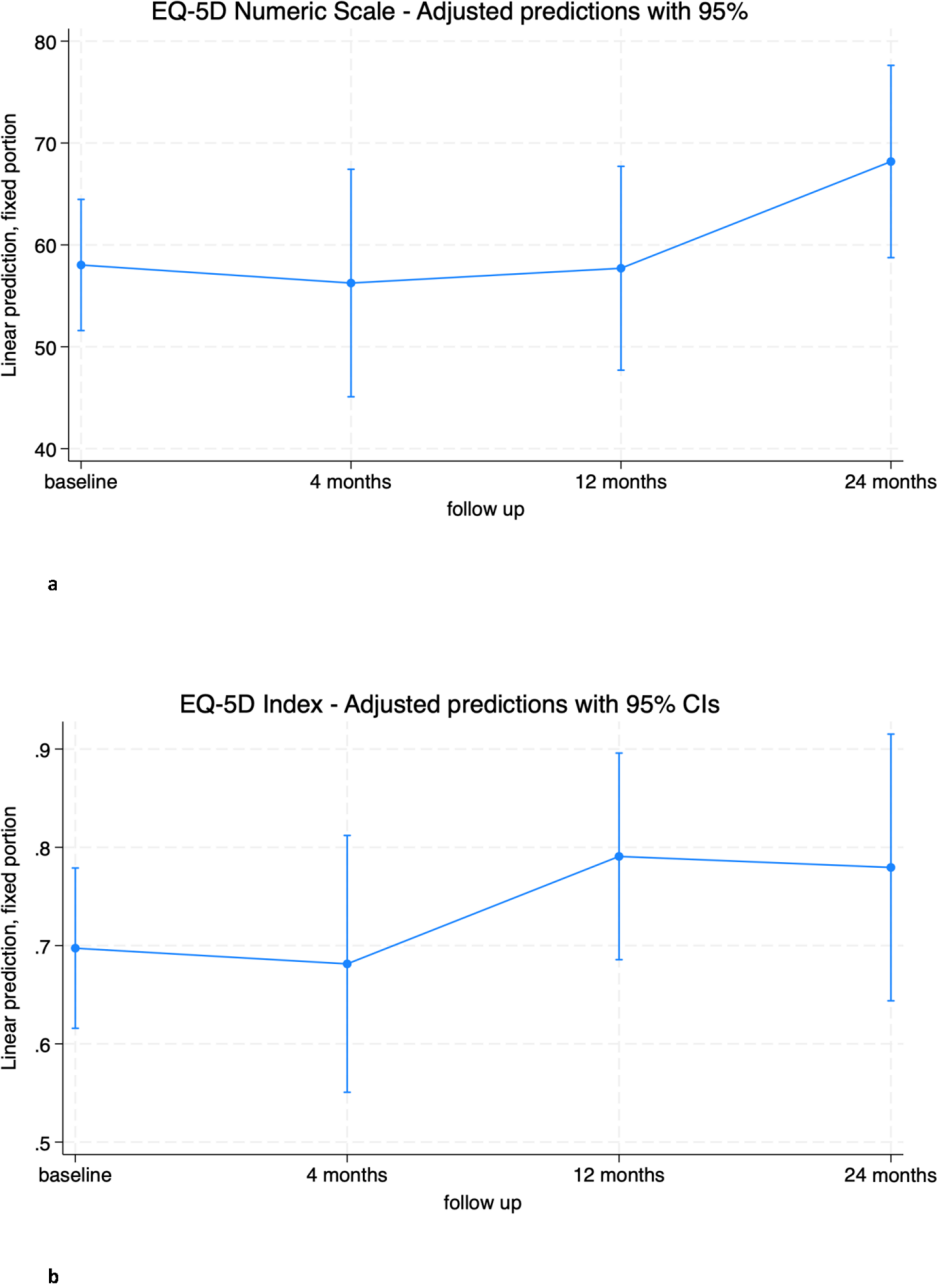
**(A)** EQ-5D Numeric Scale trend in overall population. **(B)** EQ-5D Index trend in overall population.

Considering all patients treated with en bloc resection, no significant differences were found between baseline and follow up times for EQ-5D-3L Numeric Scale and Index (**Figures 2A, B**; **Supplementary Material Tables 1**, **2**). Nonetheless, an increase in both scores between baseline and 24-months follow-up is appreciable.

The mental component SF-36 SMC showed a significant improvement at 12 months (6.2 [0.6; 11.8], p=0.028). This improvement was maintained at 24 months from baseline, although it did not reach statistical significance (5.7 [-0.34; 11.8], p=0.065).

**Figure 3:**
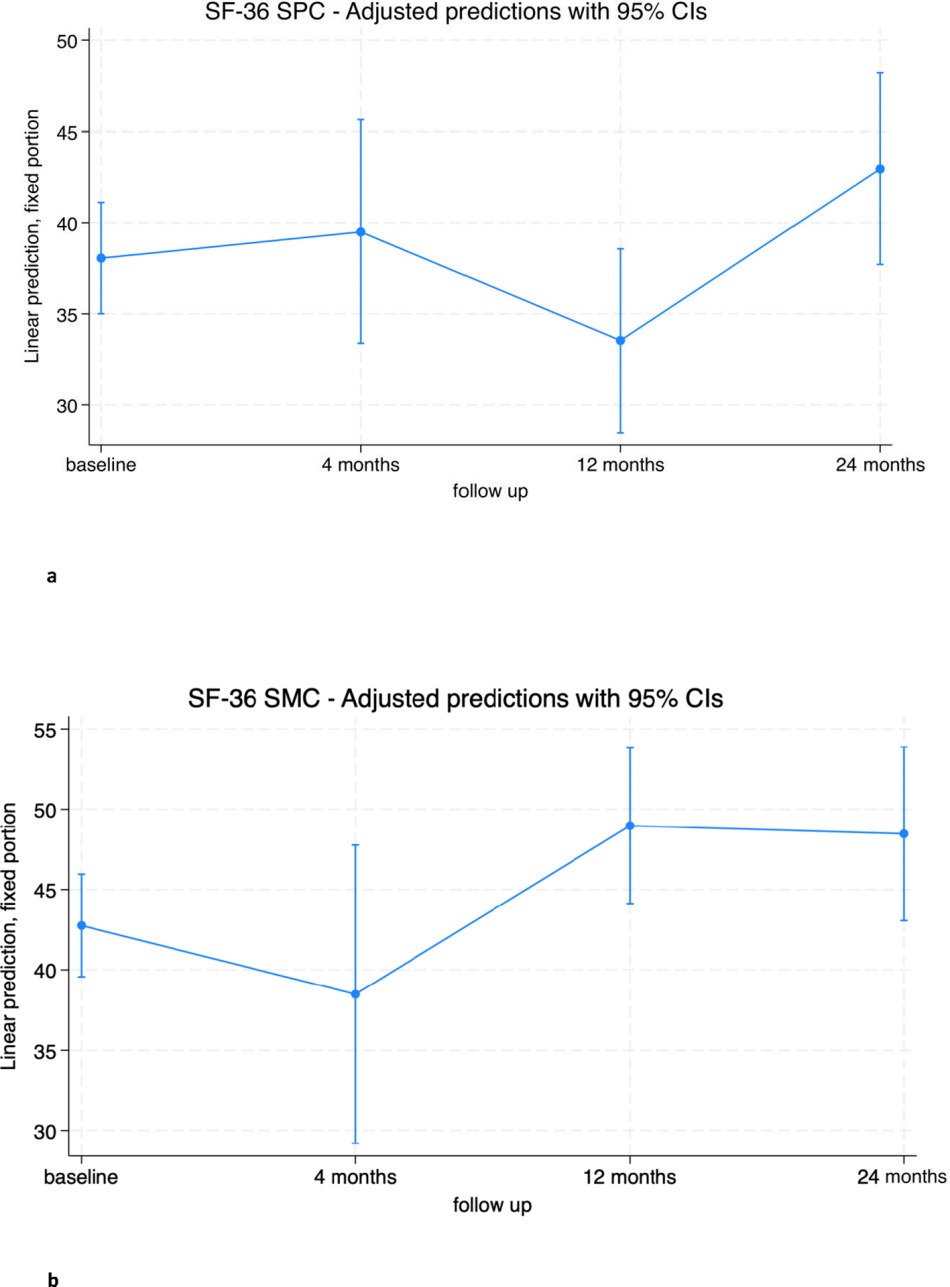
**(A)** SF-36 Standardized Physical Component (SPC) trend in overall population. **(B)** SF-36 Standardized Mental Component (SMC) trend in overall population.

The physical component SF-36 SPC did not change significantly from baseline at any of the follow-up time points, although a mean increase of almost 5 points from baseline was observed at 24 months (4.9 [-0.2; 10.0], p=0.061). Both scores reached the threshold of clinically important minimal differences but did not reach statistical significance (**Figures 3A, B**; **Supplementary Materials Tables 3**, **4**) (31, 32).

### Impact of baseline covariates on outcomes

For subsequent analyses, baseline covariates were added to the mixed model. These variables included age, sex, location of the primary spinal tumor, histotype, previous surgery, and number of vertebral levels removed (**Table 3**). Gender and previous surgery were found to have no significant impact on QOL in the cohort of patients treated with en bloc resection.

**Table 3:** Covariates for analyses on HRQOL.

*baseline covariates*							*time-dependent covariates*	
	Age	Gender	Localization	Histotype	Vertebral levels removed	Previous surgery	Adverse events	Asia score
EQ5D								
*** Numeric Scale***	**0.0214**	0.4469	0.7417	0.3210	0.1683	0.7056	**0.0034**	0.2728
*** Index***	**0.0156**	0.9113	0.3026	0.2314	**0.0348**	0.4698	**0.0001**	0.6215
SF36								
*Physical functioning*	**0.0021**	0.8354	0.8816	**0.0107**	**0.0138**	0.3485	**0.0176**	**0.0142**
*Role-physical*	0.4049	0.3272	0.0733	0.7760	0.7204	0.1219	0.1451	**0.0285**
*Bodily pain*	0.3247	0.5179	**0.0075**	0.4206	0.1182	0.4677	**0.0221**	0.1063
*General health*	0.0657	0.4957	**0.0123**	0.1804	0.7508	0.5143	0.1116	**0.0444**
*Vitality*	0.0927	0.1049	**0.0142**	0.5320	0.5299	0.2871	**0.0096**	0.3651
*Social functioning*	**0.0067**	0.3811	0.2975	0.0628	**0.0469**	0.7435	**0.0108**	**0.0369**
*Role-emotional*	**0.0093**	0.4540	0.8333	0.2773	0.3818	0.0810	**0.0000**	0.5833
*Mental health*	0.1849	0.1348	**0.0090**	0.4232	0.5729	0.1334	**0.0000**	0.5819
*** SFC***	**0.0015**	0.4031	**0.0000**	0.2172	0.0721	0.2239	0.1463	**0.0000**
*** SMC***	0.0798	0.5049	0.1616	0.4172	0.6479	0.4359	**0.0000**	0.8130
**NRS**	**0.0279**	0.9270	0.3503	0.4179	0.4275	0.2706	**0.0340**	**0.0474**

Bold values highlight the variables reported in each column.

**Figure 4:**
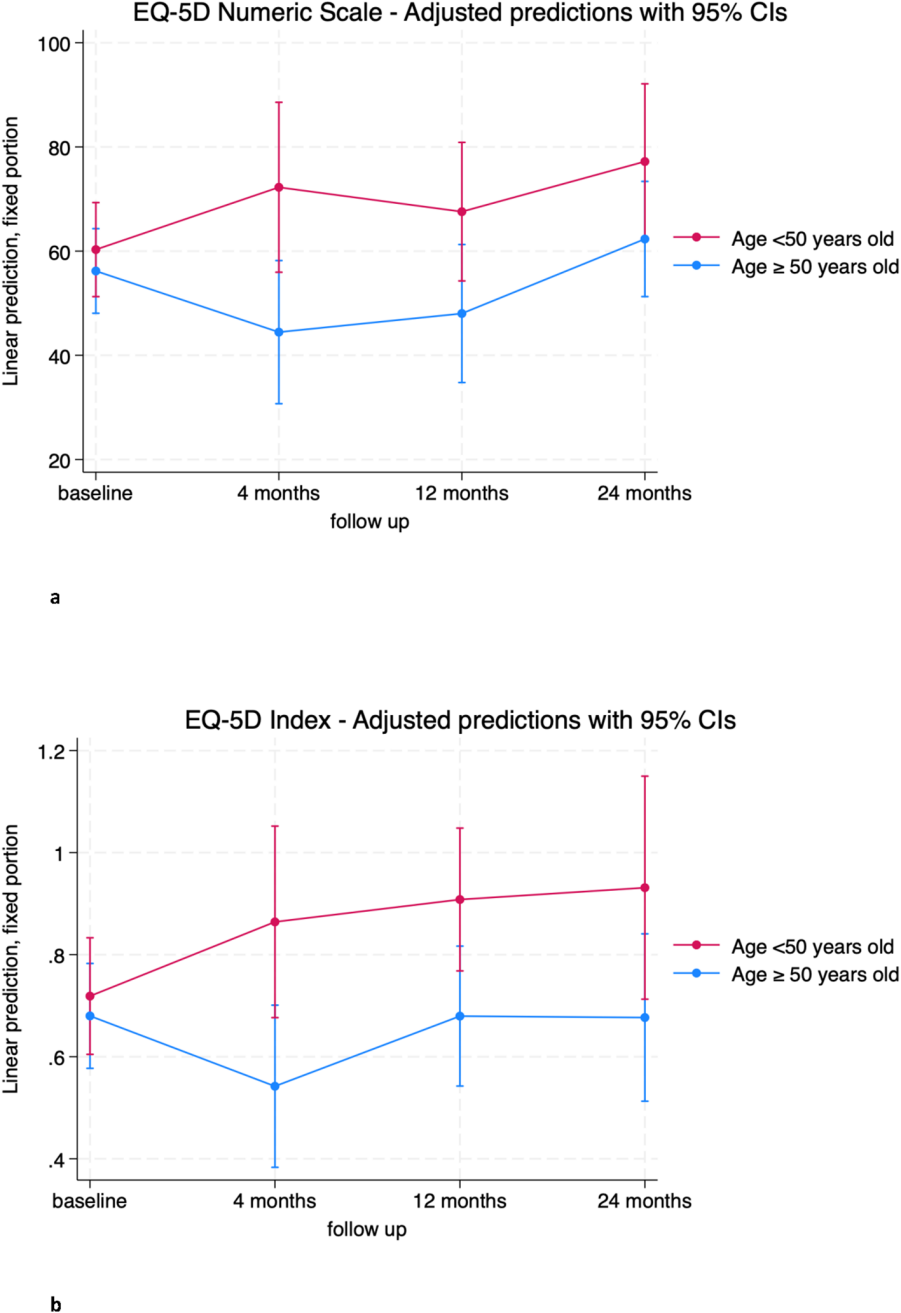
**(A)** EQ-5D Numeric Scale trends in patients over (blue) and below (red) 50 years old. **(B)** EQ-5D Index trends in patients over (blue) and below (red) 50 years old.

Age emerged as a significant factor influencing both EQ-5D-3L and SF-36 scores. The model indicates that for both the EQ-5D numeric scale and index, patients over 50 years of age are likely to have significantly worse outcomes at 4 and 12 months compared to younger patients (**Figures 4A, B**; **Tables V and VI (Supplementary Materials)**.

**Figure 5:**
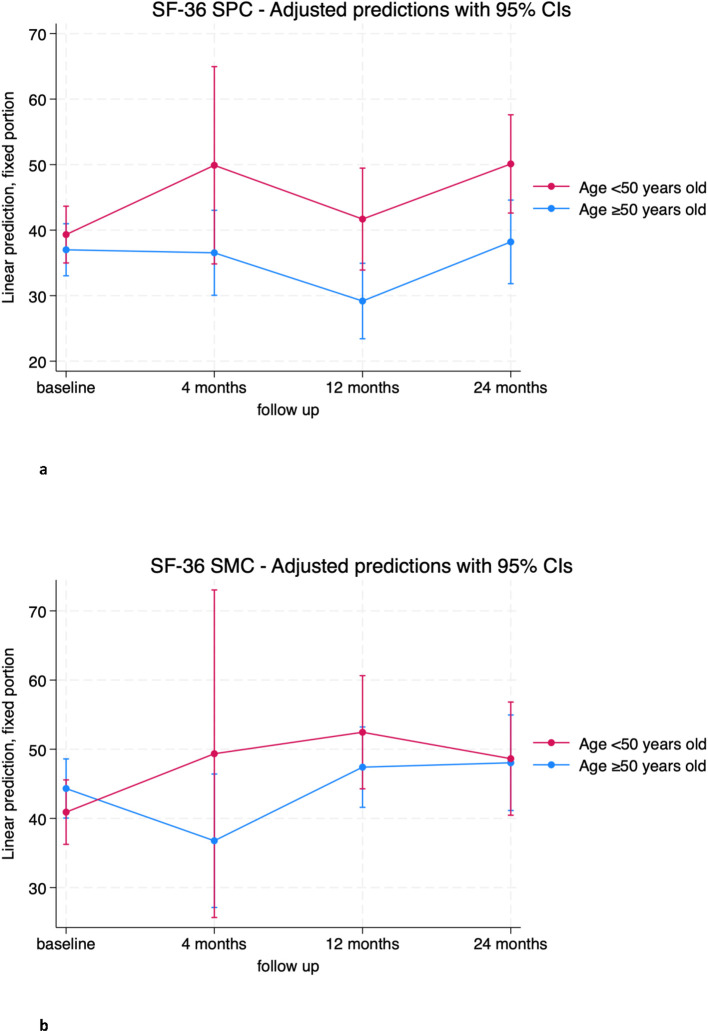
**(A)** SF-36 Standardized Physical Component (SPC) trends in patients over (blue) and below (red) 50 years old. **(B)** SF-36 Standardized Mental Component (SMC) trends in patients over (blue) and below (red) 50 years old.

Similar trends were observed for the physical component SF-36 SPC, with older patients scoring on average about 12 points lower than younger patients at 12 and 24 months (**Figure 5A**; **Supplementary Table 7**). No significant differences were observed for the SF-36 SMC (**Figure 5B**; **Supplementary Table 8**).

**Figure 6:**
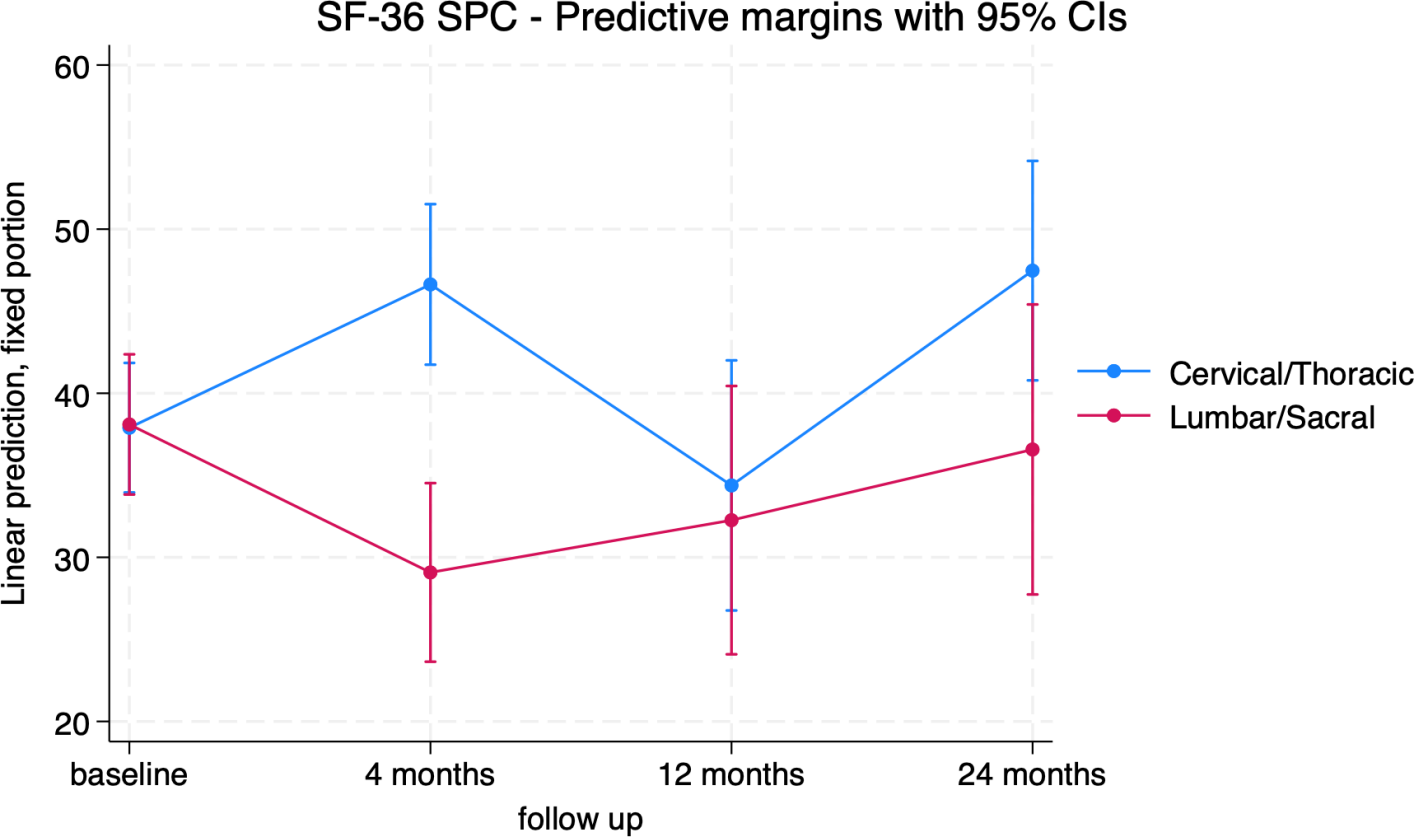
SF-36 Standardized Physical Component (SPC) trends in patients treated for Cervical/Thoracic (blue) and Lumbar/Sacral tumors (red).

The location of the primary spinal tumor appeared to have a significant impact on the standardized physical component of the SF-36. Indeed, patients with cervical and thoracic tumors showed a statistically significant mean improvement at 4 and 24 months from baseline (+8.7 [5.4; 12.0], p<0.001 and 9.6 [4.0; 15.2], p=0. 001), while patients with lumbar and sacral tumors had worse outcomes than those with cervical and thoracic tumors, particularly evident at 4 months where the difference in mean score between the two groups was -17.54 [-24.9; -10.2] (p < 0.001) (**Figure 6**; **Supplementary Table 9**). EQ-5D-3L results were not affected by tumor location.

The number of vertebral levels surgically removed indicates the invasiveness of the surgical treatment in relation to the extent of the tumor and resulted in an impairment of HRQOL.

**Figure 7:**
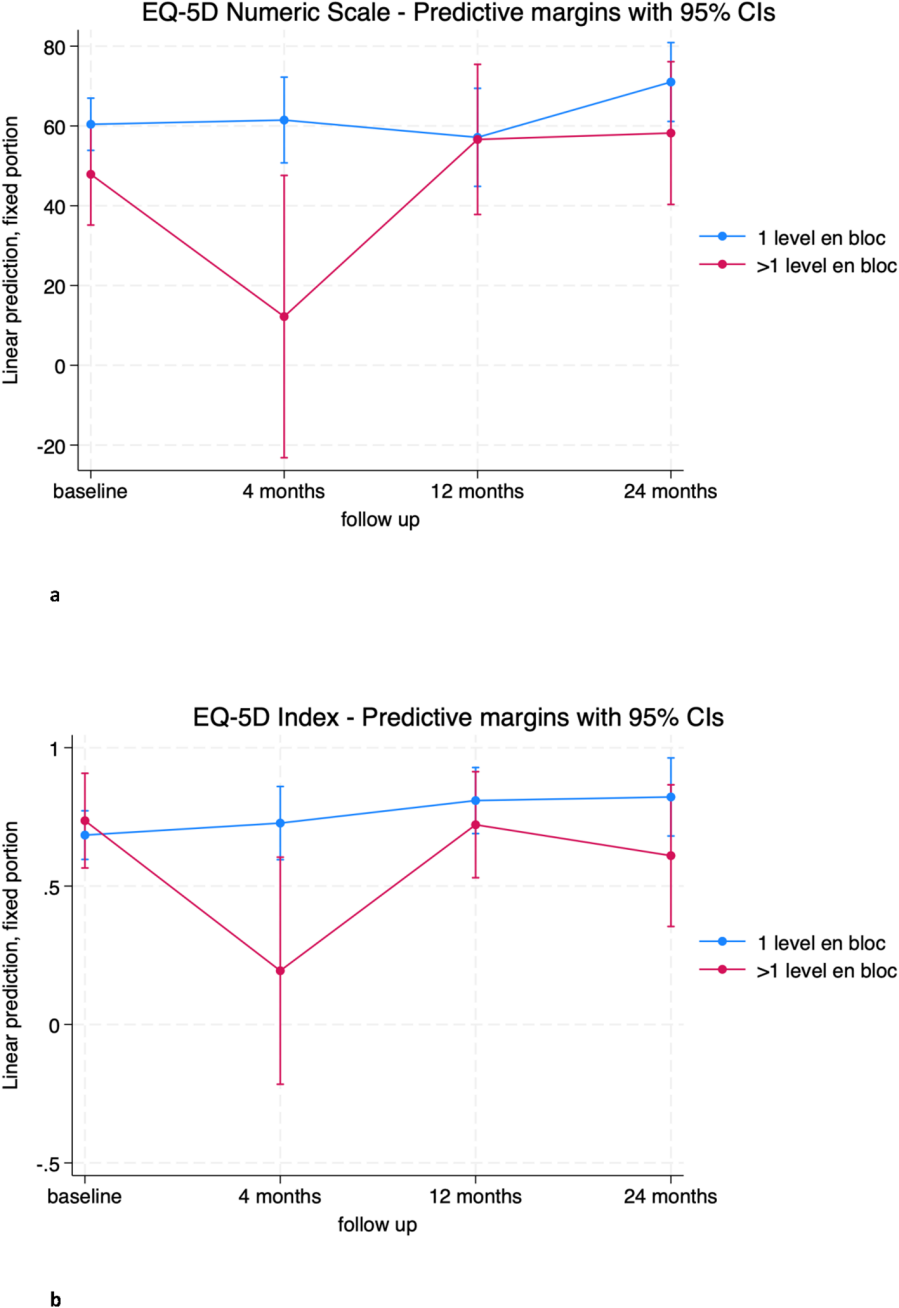
**(A)** EQ-5D Numeric Scale trends in patients treated with the en bloc resection of 1 level (blue) and >1 level (red). **(B)** EQ-5D Index trends in patients treated with the en bloc resection of 1 level (blue) and >1 level (red).

The group that underwent a single level en bloc resection consistently showed an increase in EQ-5D-3L index score from baseline at 12 months and also at the limit of significance at 24 months (0.120 [0.003; 0.247], p=0.045 and 0.138 [-0.005; 0.281], p=0.059, respectively). In contrast, patients who underwent en bloc resection of more than one level experienced a decrease in the EQ-5D-3L index at 4 months compared to the single level group (-0.53 [-0.964; -0.102], p=0.015) (**Figure 7B**; **Supplementary Table 10**). A similar result was found at 4 months on the EQ-5D-3L numeric scale (**Figure 7A**; **Supplementary Tables 11**), where the group of patients with more than one level removed had a mean score of -49.3 ([-86.2; -12.3], p = 0.009) compared to the group of patients with only one level removed. No significant differences were found between the two groups at 12 and 24 months.

### Impact of time-dependent covariates on outcomes

**Figure 8:**
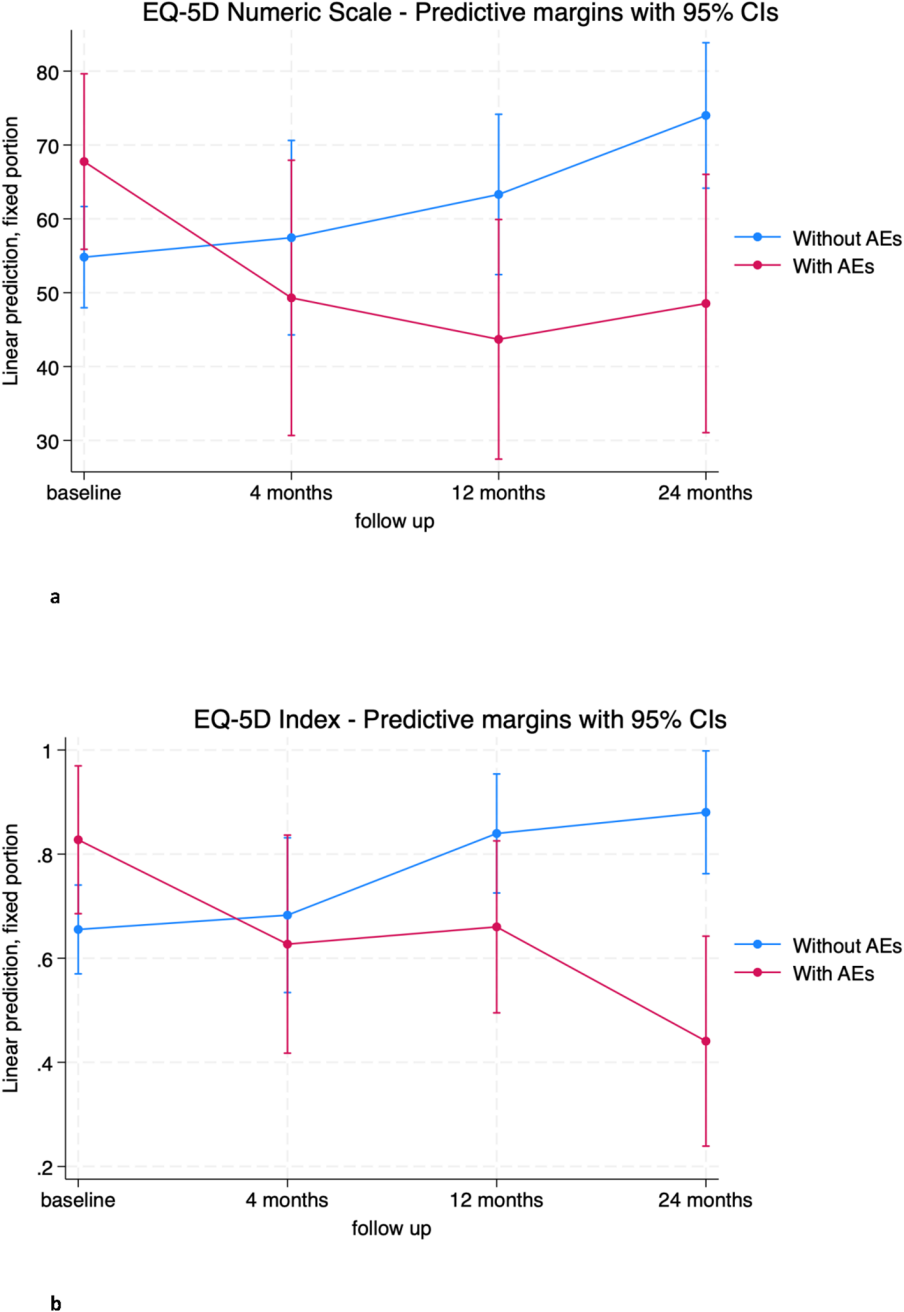
**(A)** EQ-5D Numeric Scale trends in patients without adverse event (blue) and with adverse event (red). **(B)** EQ-5D Index trends in patients without adverse event (blue) and with adverse event (red).

Complications significantly influenced quality of life scores. Both EQ-5D scores were significantly affected by the occurrence of surgical complications during follow-up (**Table 3**). In particular, patients who experienced complications during follow-up had statistically worse mean EQ-5D numeric scale scores than patients without complications at the 12- and 24-month visits (-19.62 [-38.89; -0.34], p=0.046 and -25.46 [-45.41; -5.52], p=0.012, respectively) (**Figure 8A**; **Supplementary Table 12**). The EQ-5D-3L index showed a similar trend, with a mean worsening impact of complications on patient outcome at 24 months of -0.44 ([-0.66; -0.21], p<0.001) (**Figure 8B**; **Supplementary Tables 13**).

**Figure 9:**
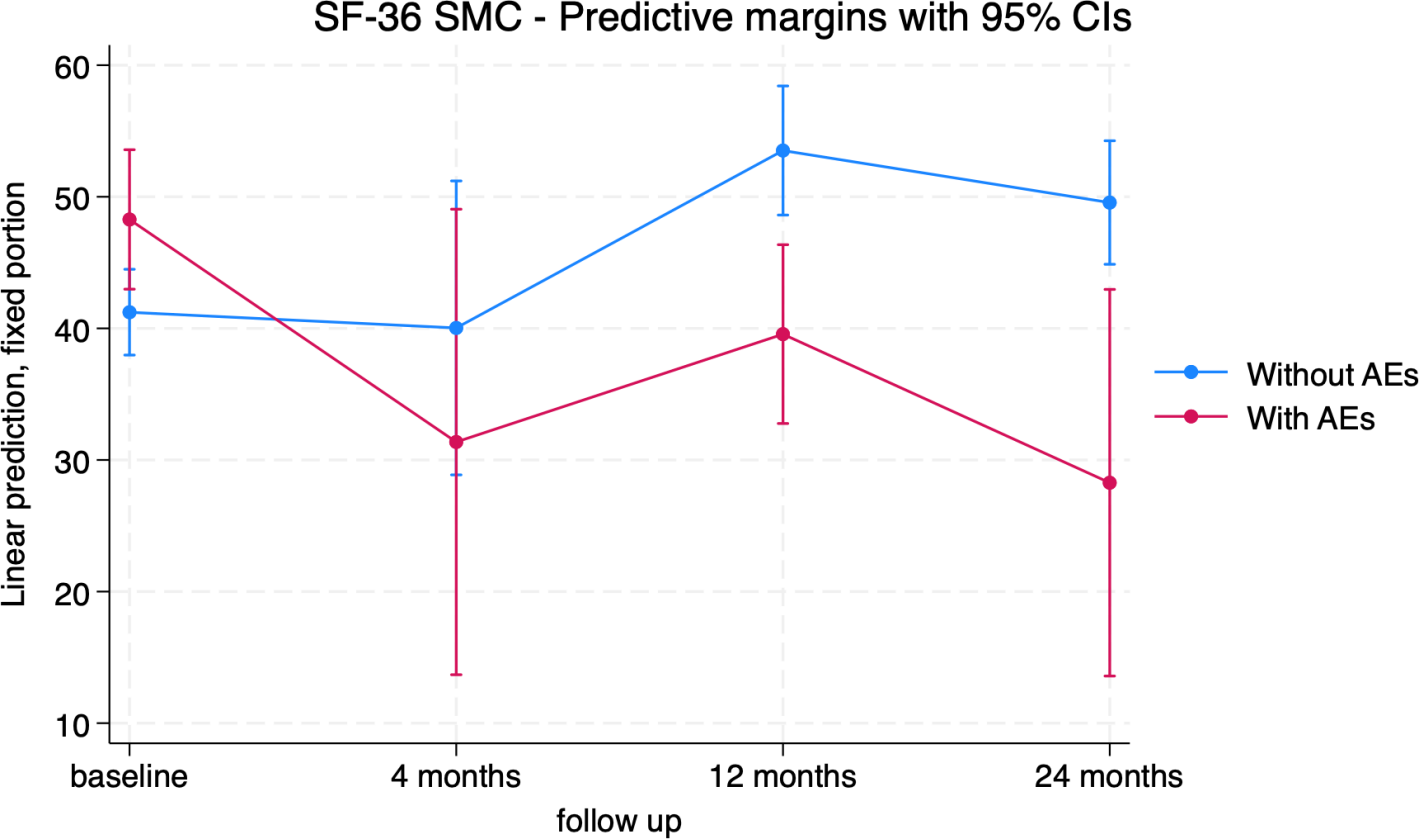
SF-36 Standardized Mental Component (SMC) trends in patients without adverse event (blue) and with adverse event (red).

Results for the SF-36 were similar. Complications (AEs) had a significant impact on most items of the SF-36 (**Table 3**). Specifically, on the SF-36 SMC, the comparison of patients with complications to patients without complications shows that the former group has significantly lower mean scores than the latter group at 12 and 24 months (-13.95 [-22.15; -5.75], p<0.001 and -21.29 [-36.62; -5.95], p=0.007, respectively) (**Figure 9**; **Supplementary Table 14**).

**Figure 10:**
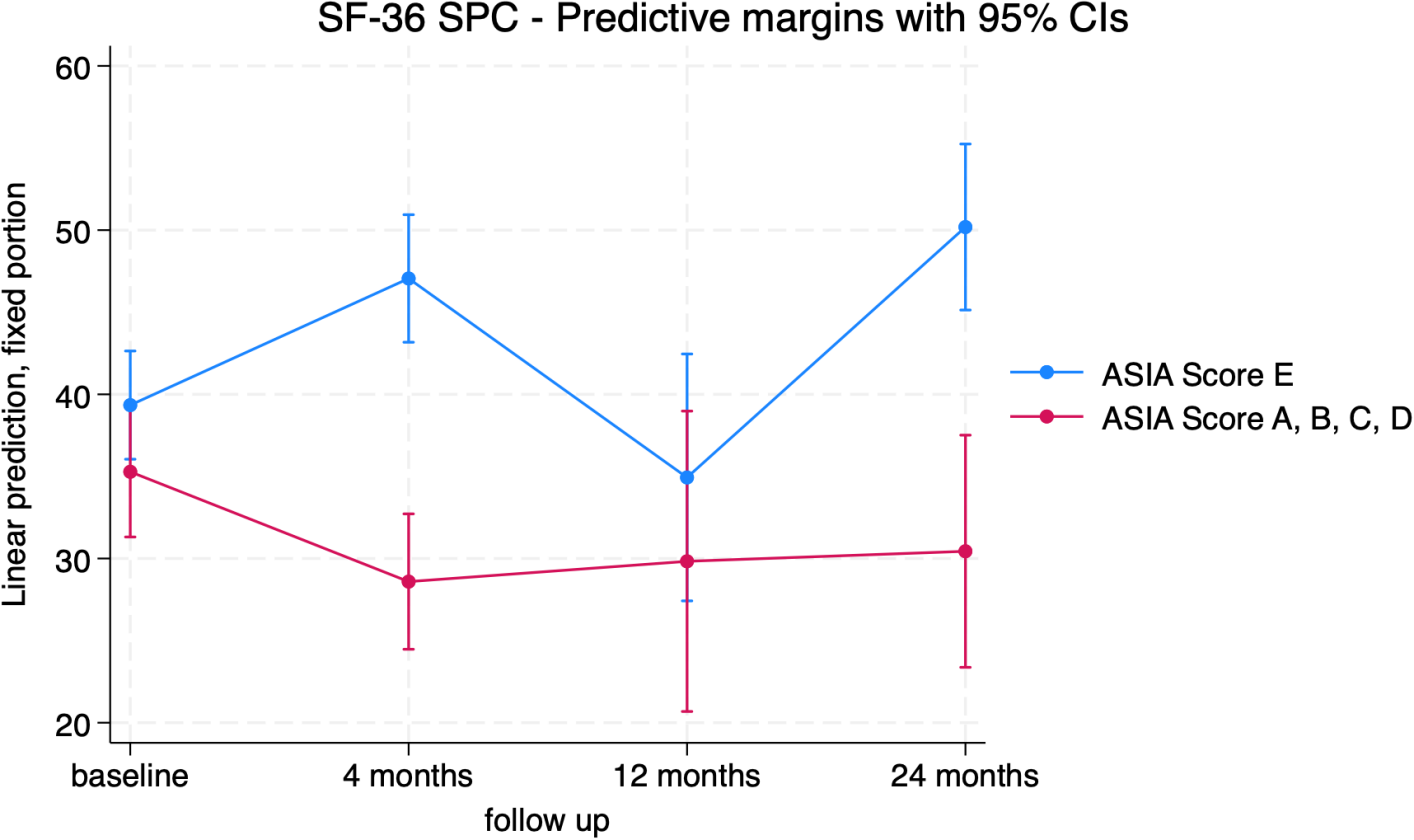
SF-36 Standardized Physical Component (SPC) trends in patients with (red) and without (blue) neurological impairment.

Regarding the SF-36 standardized physical component (PCS), patients with SCI had significantly lower mean scores than patients without SCI at the 4-month and 24-month follow-up (-18.46 [-22.19], p < 0.001 and -19.75 [-27.35; -12.13], p < 0.001, respectively) (**Figure 10**; **Supplementary Table 15**).

Similar findings were obtained for the EQ-5D-3L “Daily Activities” item and for the physical activity items of the SF-36.

### Pain

**Figure 11:**
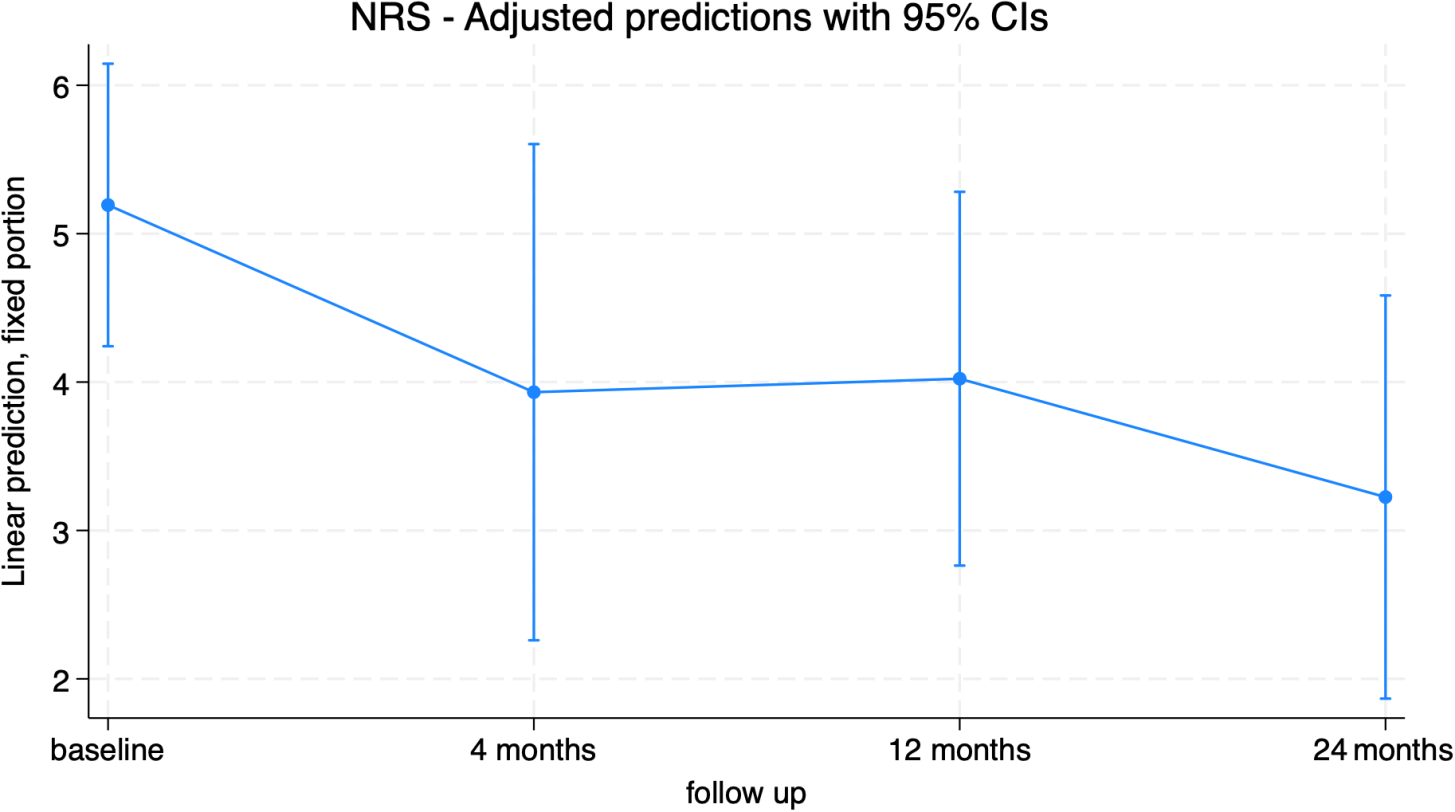
NRS trend in overall population.

**Figure 12:**
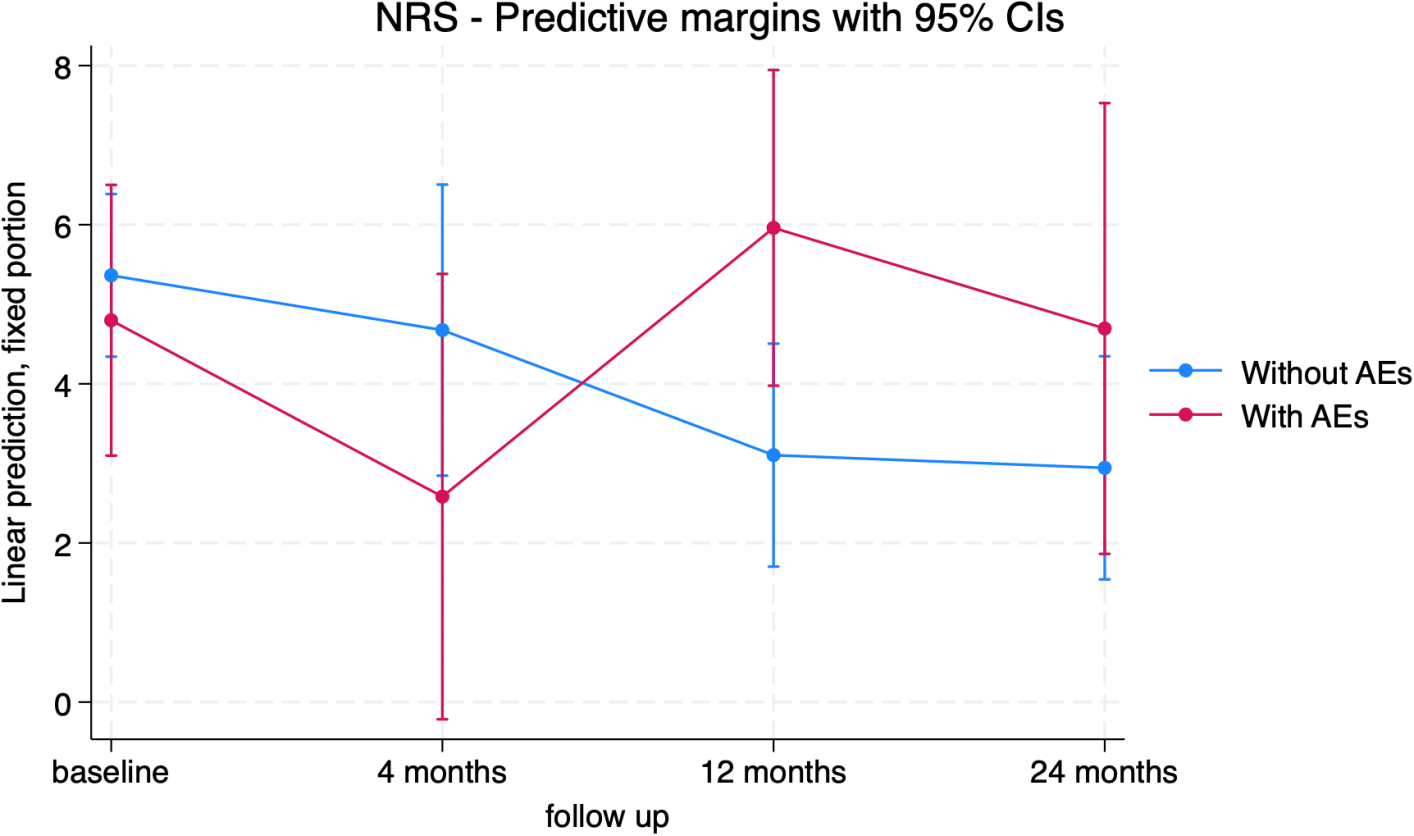
NRS trends in patients without adverse event (blue) and with adverse event (red).

The Numeric Rating Scale (NRS) was used as a dependent variable in the mixed model (**Figure 11**; **Supplementary Table 16**). Pain levels decreased after surgery, with a significant reduction at 24 months (-1.96[-3.39; -0.54], p=0.007) compared to baseline (consistent with the pain item in the EQ-5D). Significant differences were found between the groups with and without adverse events, registering with significantly higher pain scores at 12 months in the group who suffered from AEs (**Figure 12**; **Supplementary Table 17**).

## Discussion

Our findings indicate that HRQOL was not impaired by the surgery in the long-term. Conversely, the main outcome measurements (respectively Numeric Scale and Index for EQ-5D and SFC and SMC for SF-36) registered a clinically relevant improved HRQOL, even if not always statistically significant, due to the small numbers of patients at the follow-up time points. These trends show that patients generally felt well after surgery and in particular the mental health related sub-scores showed significant improvement, yet from 4-month visit. The absolute SF-36 SMC difference value between baseline and 24-month visit is higher than 5, considered as the minimal clinical important difference settled for patients with orthopaedical oncologic conditions (31). This remains an aggressive approach and some patients may experience an impairment of their activity of daily life (ADL), which is significant at 4-month follow up compared to baseline (EQ-5D-3L “Usual activities”). This finding can be confirmed observing the significant reduction of the Index at the same visit in patients who underwent more than 1 level en bloc resection compared to the single level group.

Overall, this intervention did not impair quality of life. Some patients experienced improvements in certain health domains, and, overall, they reported significantly less pain at the 24-month follow-up.

Quality of life in patients surgically treated for metastatic spine disease has been more extensively investigated (33–35), because in these patients the intent of surgical treatment is the improvement of physical function and quality of life. Some studies have addressed the HRQOL for specific tumors or surgical procedure (36–40); only one study focused on en bloc resection, comparing it with other approaches (such as radiotherapy alone) and evaluating the outcomes relative to the general population; unfortunately, no HRQOL data were available at baseline (41). As this surgery is considered in many cases the best option to obtain local control of disease regardless of the functional outcomes, the absence of specific medium-term outcome studies for patients who received en bloc resection makes it difficult to predict after-surgery status and create evidence for a patient-centered approach.

Our results concerning the effect of baseline patients’ characteristics on the trend of outcome, indicate that gender, as expected, has no impact on any of the scores or sub-scores, as well as histotype and previous surgery. On the contrary the surgical approach played an important role. Specifically, for the histotype variable, the reduction of mortality and recurrences in malignancies tends to flatten an expected difference with the benign tumors’ group.

Some other variables proved to have an impact on quality of life. The role of age seems to be more evident in the early (EQ-5D) and in the later post operative time (SF-36), when patient above >50 years exhibit significantly worse QOL. Despite the two age groups had similar level of EQ-5D and SF-36 at baseline, younger patients had better outcomes. Further studies are needed to elucidate the impact of other age-related factors (4).

Localization played a significant role. Patients treated with en bloc resection for cervical and thoracic primary tumor of the spine had consistently better HRQOL outcomes at 4-month visit compared to lumbar and sacral patients. It is noteworthy that patients undergoing surgical treatment for sacral lesions were at a higher risk of complications and adverse outcomes, including incontinence and nerve injuries, which undoubtedly impact HRQOL (42, 43). Nevertheless, these differences gradually flattened out over time and became irrelevant at 24-month visit.

Finally, our findings suggest that adverse events seem to impact on quality of life, indeed patients who experienced complications exhibited statistically poorer outcomes at 12 and 24 months. AEs appear to play a minor role in the early postoperative period when patients may prioritize the local disease control and the general oncologic outcome as the most important factors affecting their perception of health. Furthermore, early postoperative HRQOL seems once again more affected by the invasiveness of this procedure, rather than the onset of complications. The same conclusions can be drawn regarding the trend of the NRS pain scale in patients with complications. Multivariate analysis on a larger sample and tests for independence among variables could help confirm these findings.

Our previous study investigated the impact of several variables on the survival of patients treated with en bloc resection. Recurrences clearly decreased survival rates, but complications were found to have no additional effect on survival rate (4). However, our findings suggest that complications play a role in worsening HRQOL during follow up period.

The primary limitation of this study is the small sample size, which restricted our ability to detect significant differences between patient subgroups and prevented us from drawing robust clinical conclusions. A more consistent and systematic collection of HRQOL assessment tools, along with a longer follow-up period, could help confirm and generalizing our early observations.

A further limitation is the heterogeneity of diagnoses among these patients. However, this must be viewed in the context of the rarity of primary bone tumors of the spine and of this complex surgical procedure. It should be noted that no significant differences were reported between patients affected by benign or malignant tumors concerning HRQOL scores.

On the other hand, one of the key strengths of this analysis is the homogeneity in the surgical approach, as all procedures were performed by the same team of surgeons, at the same institution, using the same technique.

## Conclusion

Our preliminary results suggest that patients treated with en bloc resection for primary spinal tumors, with first aim of ensuring better survival rates, typically do not experience a diminished HRQOL and in some cases it is registered a slight to moderate postoperative improvement compared to their preoperative perceived health status. These results are confirmed by our recent study analyzing the impact of en bloc resection with adequate negative margins *versus* intralesional resection *plus* adjuvant proton beam/carbon ion therapy on local control and overall survival in patients with mobile spine chordomas (17). We observed no significant differences in Overall Survival (OS) and Local Recurrence Disease Free Survival (LRDFS) between the two groups of patients, while the wide margins group had better quality of life scores at the last follow-up, with a significant improvement in the same group comparing the last follow-up with baseline.

While en bloc resection offers improved survival, it is associated with high rates of adverse events. The occurrence of complications, along with older age, was found to be the most significant factor negatively impacting HRQOL in these patients. Further studies with a bigger sample size could confirm our findings and focus on identifying which adverse events are of greater significance and what the associated risk factors are, not only to mitigate their impact on HRQOL, but also because these same risk factors may influence survival. A comprehensive understanding could lead to the development of new surgical guidelines for patients affected by spinal tumors, who may experience both lower survival rates and diminished HRQOL.

## Data Availability

The original contributions presented in the study are included in the article/**Supplementary Material**. Further inquiries can be directed to the corresponding author.
