# Supplementary material for: Health-related quality of life in patients treated with en bloc resection for primary tumors of the spine
Source: Front Oncol. 2024 Nov 20;14:1485226. doi: 10.3389/fonc.2024.1485226 (PMC11618236; doi:10.3389/fonc.2024.1485226)
Supplement: Supplementary file 1 [file Table1.docx]

**Supplementary Material**

**Tab. I EQ5D Numeric Scale**

**EQ5D Numeric Scale** Coefficient Std. err. z P>z [95% conf. interval]

visit

4 months -1.771817 6.19913 -0.29 0.775 -13.92189 10.37825

12 months -.3215628 5.660658 -0.06 0.955 -11.41625 10.77312

24 months 10.15552 5.400344 1.88 0.060 -.4289578 20.74

_cons 58.02632 3.284645 17.67 0.000 51.58853 64.4641

**Tab. II EQ5D Index**

**EQ5D Index** Coefficient Std. err. z P>z [95% conf. interval]

visit

4 months -.0159946 .0675258 -0.24 0.813 -.1483427 .1163535

12 months .0933993 .0547153 1.71 0.088 -.0138408 .2006393

24 months .082138 .0700728 1.17 0.241 -.0552022 .2194781

_cons .6973684 .0416128 16.76 0.000 .6158088 .778928

**Tab. III SF36 SPC**

**SF36 SPC** Coefficient Std. err. z P>z [95% conf. interval]

visit

4 months 1.444596 3.069973 0.47 0.638 -4.572441 7.461633

12 months -4.528875 2.502168 -1.81 0.070 -9.433035 .3752842

24 months 4.89778 2.610043 1.88 0.061 -.2178106 10.01337

_cons 38.06357 1.558384 24.43 0.000 35.0092 41.11795

**Tab.IV SF36 SMC**

**SF36 SMC** Coefficient Std. err. z P>z [95% conf. interval]

visit

4 months -4.278502 4.937917 -0.87 0.386 -13.95664 5.399636

12 months 6.213826 2.834384 2.19 **0.028** .6585353 11.76912

24 months 5.710121 3.088726 1.85 0.065 -.3436699 11.76391

_cons 42.7597 1.631145 26.21 0.000 39.56271 45.95668

**Tab. V age for EQ-5D Numeric Scale**

**EQ-5D NumScale** Coefficient Std.err. z P>z [95% conf. interval]

visit

(>=50) 4 months -11.7422 7.688527 -1.53 0.127 26.81144 3.327033

(>=50) 12 months -8.164818 7.460114 -1.09 0.274 22.78637 6.456737

(>=50) 24 months 6.135968 6.463003 0.95 0.342 6.531286 18.80322

Post-hoc

Contrast Std. err. [95% conf. interval] P>chi2

(18-49 vs >=50) baseline 4.103641 6.196466 -8.04121 16.24849 0.5078

(18-49 vs >=50) 4 months 27.80809 10.88032 6.483057 49.13313 **0.0106**

(18-49 vs >=50) 12 months 19.5476 9.576837 .7773486 38.31786 **0.0412**

(18-49 vs >=50) 24 months 14.86765 9.473565 -3.700197 33.4355 0.1166

**Tab. VI age for EQ-5D index**

**EQ5D index** Coefficient Std. err. z P>z [95% conf. interval]

visit

(>=50) 4 months -.1378475 .081382 -1.69 0.090 -.2973533 .0216584

(>=50) 12 months -.0003839 .0703102 -0.01 0.996 -.1381893 .1374215

(>=50) 24 months -.0032025 .0839691 -0.04 0.970 -.1677789 .1613739

Post-hoc

Contrast Std. err. [95% conf. interval] P>chi2

(18-49 vs >=50) baseline .0388235 .0783871 -.1148123 .1924593 0.6204

(18-49 vs >=50) 4 months .322113 .1254624 .0762113 .5680148 **0.0102**

(18-49 vs >=50) 12 months .2284636 .0999554 .0325546 .4243726 **0.0223**

(18-49 vs >=50) 24 months .2545094 .1393905 -.018691 .5277098 0.0679

**Tab. VII age for sf36 SPC**

**SF36 SPC** Coefficient Std. err. z P>z [95% conf. interval]

visit

(>=50) 4 months -.462333 3.070129 -0.15 0.880 -6.479676 5.55501

(>=50) 12 months -7.826534 2.665318 -2.94 0.003 -13.05046 -2.602606

(>=50) 24 months 1.200676 3.011164 0.40 0.690 -4.701097 7.102449

Post-hoc

Contrast Std. err. [95% conf. interval] P>chi2

(18-49 vs >=50) baseline 2.316084 2.999183 -3.562206 8.194374 0.4400

(18-49 vs >=50) 4 months 13.3565 8.364208 -3.037047 29.75005 0.1103

(18-49 vs >=50) 12 months 12.50923 4.938578 2.829799 22.18867 **0.0113**

(18-49 vs >=50) 24 months 11.90132 5.023335 2.055768 21.74688 **0.0178**

**Tab. VIII age for SF36 SMC**

**SF36 SMC** Coefficient Std. err. z P>z [95% conf. interval]

visit

(>=50) 4 months -7.55316 5.196312 -1.45 0.146 -17.73775 2.631425

(>=50) 12 months 3.088843 3.400592 0.91 0.364 -3.576194 9.753881

(>=50) 24 months 3.726604 3.896962 0.96 0.339 -3.911301 11.36451

Post-hoc

Contrast Std. err. [95% conf. interval] P>chi2

(18-49 vs >=50) baseline -3.415248 3.226062 -9.738213 2.907717 0.2898

(18-49 vs >=50) 4 months 12.58218 13.04481 -12.98518 38.14953 0.3348

(18-49 vs >=50) 12 months 5.044404 5.119028 -4.988707 15.07751 0.3244

(18-49 vs >=50) 24 months .5906434 5.461386 -10.11348 11.29476 0.9139

**Tab. IX Localization for SF36 SPC**

**SF36 SPC** Coefficient Std. err. z P>z [95% conf. interval]

Cervical/Thoracic (C/T) .2009991 2.959243 0.07 0.946 -5.59901 6.001008

visit

4 months 8.731695 1.676566 5.21 **0.000** 5.445687 12.0177

12 months -3.516758 3.417581 -1.03 0.303 -10.21509 3.181577

24 months 9.571299 2.861941 3.34 **0.001** 3.961997 15.1806

ageatsurgery -.0324735 .0825848 -0.39 0.694 -.1943368 .1293898

_cons 39.58887 4.509177 8.78 0.000 30.75105 48.42669

Post-hoc

Contrast Std. err. [95% conf. interval] P>chi2

(C/T vs L/S) baseline .2009991 2.959243 -5.59901 6.001008 0.9458

C/T vs L/S) 4 months -17.54976 3.732806 -24.86592 -10.23359 **0.0000**

C/T vs L/S) 12 months -2.115959 5.700296 -13.28833 9.056416 0.7105

C/T vs L/S) 24 months -10.89633 5.649944 -21.97001 .1773603 0.0538

**Tab. X Enbloc level for EQ-5D Numeric Scale**

**EQ-5D NumScale** Coefficient Std. err. z P>z [95% conf. interval]

1.enbloc -12.53118 7.297926 -1.72 0.086 -26.83486 1.772487

visit

(1 lev) 4 months 1.066488 6.210416 0.17 0.864 -11.1057 13.23868

(1 lev) 12 months -3.275192 6.910294 -0.47 0.636 -16.81912 10.26874

(1 lev) 24 months 10.60098 5.82247 1.82 0.069 -.8108506 22.01281

Post-hoc

Contrast Std. err. [95% conf. interval] P>chi2

(1 lev vs >1 lev) baseline -12.53118 7.297926 -26.83486 1.772487 0.0860

(1 lev vs >1 lev) 4 months -49.27118 18.86675 -86.24934 -12.29303 **0.0090**

(1 lev vs >1 lev) 12 months -.5191133 11.46687 -22.99377 21.95554 0.9639

(1 lev vs >1 lev) 24 months -12.79943 10.43214 -33.24605 7.647182 0.2199

**Tab. XI Enbloc level for EQ-5D index**

**EQ5D Index** Coefficient Std. err. z P>z [95% conf. interval]

1.enbloc .0521763 .0980507 0.53 0.595 -.1399994 .2443521

visit

(1 lev) 4 months .0433532 .068652 0.63 0.528 -.0912023 .1779087

(1 lev) 12 months .1247958 .0621545 2.01 **0.045** .0029752 .2466163

(1 lev) 24 months .1377102 .0730004 1.89 0.059 -.005368 .2807883

Post-hoc

Contrast Std. err. [95% conf. interval] P>chi2

(1 lev vs >1 lev) baseline .0521763 .0980507 -.1399994 .2443521 0.5946

(1 lev vs >1 lev) 4 months -.5333808 .2199225 -.964421 -.1023406 **0.0153**

(1 lev vs >1 lev) 12 months -.0873538 .115098 -.3129417 .1382341 0.4479

(1 lev vs >1 lev) 24 months -.2119745 .1490953 -.504196 .0802469 0.1551

**Tab. XII AEs for EQ-5D Numeric Scale**

**EQ-5D NumScale** Coefficient Std. err. z P>z [95% conf. interval]

AENO0SI1 12.94404 6.864591 1.89 0.059 -.510307 26.39839

visit

(no AEs) 4 months 2.633432 7.141852 0.37 0.712 -11.36434 16.6312

(no AEs) 12 months 8.489177 5.993379 1.42 0.157 -3.25763 20.23598

(no AEs) 24 months 19.18676 5.486314 3.50 **0.000** 8.433788 29.93974

Post-hoc

Contrast Std. err. [95% conf. interval] P>chi2

(AEs vs no AEs) baseline 12.94404 6.864591 -.510307 26.39839 0.0593

(AEs vs no AEs) 4 months -8.148991 11.50791 -30.70409 14.40611 0.4789

(AEs vs no AEs) 12 months -19.61969 9.83394 -38.89386 -.3455274 **0.0460**

(AEs vs no AEs) 24 months -25.46508 10.17609 -45.40985 -5.52031 **0.0123**

**Tab. XIII AEs for EQ-5D index**

**EQ5D Index** Coefficient Std. err. z P>z [95% conf. interval]

AENO0SI1 .1723019 .080224 2.15 0.032 .0150658 .329538

visit

(no AEs) 4 months .0274808 .0787176 0.35 0.727 -.1268028 .1817644

(no AEs) 12 months .1843822 .0612754 3.01 **0.003** .0642846 .3044799

(no AEs) 24 months .2250632 .0625337 3.60 **0.000** .1024994 .347627

Post-hoc

Contrast Std. err. [95% conf. interval] P>chi2

(AEs vs no AEs) baseline .1723019 .080224 .0150658 .329538 **0.0317**

(AEs vs no AEs) 4 months -.0556903 .1274218 -.3054325 .1940519 0.6621

(AEs vs no AEs) 12 months -.179461 .0982184 -.3719656 .0130435 0.0677

(AEs vs no AEs) 24 months -.4396098 .1160484 -.6670605 -.2121591 **0.0002**

**Tab. XIV AEs for SF36 SMC**

**SF36 SMC** Coefficient Std. err. z P>z [95% conf. interval]

AENO0SI1 7.04731 3.096235 2.28 0.023 .9787999 13.11582

visit

(no AEs) 4 months -1.194783 5.785348 -0.21 0.836 -12.53386 10.14429

(no AEs) 12 months 12.28541 2.712122 4.53 **0.000** 6.969749 17.60107

(no AEs) 24 months 8.332711 2.559838 3.26 **0.001** 3.31552 13.3499

Post-hoc

Contrast Std. err. [95% conf. interval] P>chi2

(AEs vs no AEs) baseline 7.04731 3.096235 .9787999 13.11582 **0.0228**

(AEs vs no AEs) 4 months -8.667912 10.61733 -29.47749 12.14167 0.4143

(AEs vs no AEs) 12 months -13.9515 4.183549 -22.15111 -5.751899 **0.0009**

(AEs vs no AEs) 24 months -21.29027 7.826262 -36.62946 -5.951075 **0.0065**

**Tab. XV ASIA for SF36 SPC**

**SF36 SPC** Coefficient Std. err. z P>z [95% conf. interval]

ASIA E . 057913 1.733178 -2.34 **0.019** -7.454879 -.6609461

visit

4 months 7.71165 1.323955 5.82 **0.000** 5.116745 10.30655

12 months -4.404684 3.582802 -1.23 0.219 -11.42685 2.617478

24 months 10.8451 2.121663 5.11 **0.000** 6.686721 15.00349

ageatsurgery -.0134806 .0873407 -0.15 0.877 -.1846653 .1577041

_cons 40.03942 4.640001 8.63 0.000 30.94519 49.13366

Post-hoc

Contrast Std. err. [95% conf. interval] P>chi2

(ASIA <E vs E) baseline -4.057913 1.733178 -7.454879 -.6609461 **0.0192**

(ASIA <E vs E) 4 months -18.45768 1.90495 -22.19131 -14.72405 **0.0000**

(ASIA <E vs E) 12 months -5.105633 5.678661 -16.2356 6.024338 0.3686

(ASIA <E vs E) 24 months -19.74505 3.881666 -27.35298 -12.13712 **0.0000**

**Tab. XVI NRS tot**

**NRS tot** Coefficient Std. err. z P>z [95% conf. interval]

visit

4 months -1.261344 .8773068 -1.44 0.151 -2.980833 .458146

12 months -1.170564 .6780847 -1.73 0.084 -2.499585 .1584581

24 months -1.96822 .7268652 -2.71 **0.007** -3.392849 -.54359

_cons 5.193297 .4857136 10.69 0.000 4.241316 6.145278

**Tab. XVII AEs for NRS**

Contrast Std. err. [95% conf. interval] P>chi2

(AEs vs no AEs) baseline -.5644738 .9759768 -2.477353 1.348406 0.5630

(AEs vs no AEs) 4 months -2.092682 1.669814 -5.365458 1.180095 0.2101

(AEs vs no AEs) 12 months 2.856511 1.19555 .5132771 5.199746 0.0169

(AEs vs no AEs)24 months 1.751606 1.59017 -1.365069 4.868281 0.2707
